# Flipping the Classroom in Medical Student Education: Does Priming Work?

**DOI:** 10.5811/westjem.2017.8.35162

**Published:** 2017-12-05

**Authors:** Emily Rose, Paul Jhun, Matthew Baluzy, Aaron Hauck, Jonathan Huang, Jonathan Wagner, Yvette L. Kearl, Solomon Behar, Ilene Claudius

**Affiliations:** * Los Angeles County-University of Southern California Medical Center, Department of Emergency Medicine, Los Angeles, California; † University of California San Francisco, San Francisco General Hospital, Department of Emergency Medicine, San Francisco, California; ‡ Keck School of Medicine of the University of Southern California, Los Angeles, California

## Abstract

**Introduction:**

The emergency medicine (EM) clerkship curriculum at Los Angeles County + University of Southern California Medical Center includes monthly lectures on pediatric fever and shortness of breath (SOB). This educational innovation evaluated if learning could be enhanced by “priming” the students with educational online videos prior to an in-class session. Factors that impacted completion rates were also evaluated (planned specialty and time given for video viewing).

**Methods:**

Twenty-minute videos were to be viewed prior to the didactic session. Students were assigned to either the fever or SOB group and received links to those respective videos. All participating students took a pre-test prior to viewing the online lectures. For analysis, test scores were placed into concordant groups (test results on fever questions in the group assigned the fever video and test results on SOB questions in the group assigned the SOB video) and discordant groups (crossover between video assigned and topic tested). Each subject contributed one set of concordant results and one set of discordant results. Descriptive statistics were performed with the Mann-Whitney U test. Lecture links were distributed to students two weeks prior to the in-class session for seven months and three days prior to the in-class session for eight months (in which both groups included both EM-bound and non-EM bound students).

**Results:**

In the fifteen-month study period, 64% of students rotating through the EM elective prepared for the in class session by watching the videos. During ten months where exclusively EM-bound students were rotating (n=144), 71.5% of students viewed the lectures. In four months where students were not EM-bound (n=54), 55.6% of students viewed the lectures (p=0.033). Participation was 60.2% when lecture links were given three days in advance and 68.7% when links were given two weeks in advance (p=0.197). In the analysis of concordant scores, the pre-test averaged 56.7% correct, the immediate post-test averaged 78.1% correct, and the delayed post-test was 67.2%. In the discordant groups, the pretest averaged 51.9%, the immediate posttest was 67.1% and the delayed by 68.8%. In the concordant groups, the immediate post-test scores improved by 21.4%, compared with 15.2% in the discordant groups (p = 0.655). In the delayed post-test the concordant scores improved by 10.5% and discordant scores by 16.9 percent (p=0.609). Sixty-two percent of students surveyed preferred the format of online videos with in-class case discussion to a traditional lecture format.

**Conclusion:**

Immediate post-tests and delayed post-tests improved but priming was not demonstrated to be a statistically superior educational method in this study. Medical student completion of the preparatory materials for the EM rotation session increased when the students were EM-bound. Participation rates were not significantly different when given at two weeks versus three days.

## INTRODUCTION

The flipped classroom is a novel educational method recently adopted for learners at all levels including in medical school.^1^–^6^ This teaching model has students do “homework” prior to class in order to free up class time for a more interactive and engaging session. Currently, the implementation and structure of the flipped classroom is not well defined. Additionally, it requires “buy-in” and participation from the students in order to be successful. The student must invest in the method and participate in order to learn the didactic material and to have a fruitful in-class interaction. How best to effectively implement the flipped classroom in medical education remains unknown. Self-study, formal education and clinical exposure all must occur, and each area competes for the learner’s time. Education must be both effective and efficient.

The details of effective implementation of the flipped classroom are important for two reasons: (1) to obtain the “buy-in” of students to complete an assigned task prior to class; and (2) to justify the significant time investment of faculty to overhaul curriculum changes. This study sought to evaluate if “priming” prior to an in-class session improved a medical student’s knowledge base, both immediately after the session and in the form of a delayed test. This study also examined factors that influenced pre-class video viewing, which contributes to the success of implementing the flipped classroom in a clerkship rotation.

## METHODS

### Study setting and population

We performed this prospective descriptive study from May 2015 to September 2016. A total of 212 students rotated through the emergency medicine (EM) clerkship at Los Angeles County + University of Southern California Medical Center, a Level I trauma center with approximately 170,000 annual visits. Four 10-minute videos were filmed by the pediatric EM faculty, which were to be viewed prior to the in-class, case-based discussion. There were two videos on fever and two on shortness of breath (SOB) (covering laryngotracheo-bronchitis/croup and bronchiolitis). Students accessed the videos via Zaption (www.zaption.com), an online service that provides instant analytics including the date, time and number of video views, average viewing time, and percent of video watched. Students were not aware that their video views were being tracked.

### Study Protocol

Students were assigned to either the fever or SOB group by alternating an alphabetical list of rotators. All participating students took a pre-test prior to viewing the online lectures. Those in the fever group received lecture links to two fever videos and those in the SOB group received links to two videos on the topics of croup and bronchiolitis. For eight months (five of those months with EM-bound students and three with non-EM bound students), the links were sent three days prior to the in-class session. For seven months (five with EM-bound students and two with non-EM bound students) the links were sent two weeks prior to class, with two additional email reminders to complete the viewing (Table 1).

**Population Health Research Capsule**

What do we already know about this issue?

The flipped classroom is an educational innovation with a high student-satisfaction rate. Whether it enhances education and learning retention enough to justify significant curriculum changes has not yet been demonstrated.

What was the research question?

We sought to evaluate whether priming, a component of the flipped classroom, enhanced retention of material, i.e. would students test better if they were “primed” for learning by watching videos? Additionally, would EM-bound students be more likely to complete the preparatory material, and was that completion rate affected by how far in advance the videos were distributed?

What was the major finding of the study?

While priming did not appear to impact learning retention, EM-bound students were more likely to complete the preparatory materials. Advanced distribution of pre-class education videos did not lead to a statistically significant increased viewing rate.

How does this improve population health?

When teaching innovations lead to more effective medical education it translates to enhanced patient care. Additionally, successful medical education strategies could be implemented to improve patient education.

The classroom session consisted of a one-hour, case-based discussion of a febrile neonate and the management of pediatric fever across age groups, and a one-hour, case-based discussion of a three-year-old child in respiratory distress ultimately discovered to have viral laryngotracheobronchitis (croup). After participation in the in-class session, all students immediately took another test on both fever and SOB. Following the in-class session, students in the fever group received a transcript of the SOB video and students from the SOB group received a transcript of the fever video to balance the amount of information given on each topic. A third test on these topics was administered at the end of the rotation (three weeks after the immediate post-test).

Three tests were written and reviewed by three board-certified, pediatric-trained emergency physicians. Each test contained questions on the topics of pediatric fever, bronchiolitis and laryngotracheobronchitis. The three tests (A, B, C) were rotated each month to compensate for the possibility that one test may have been more difficult than the others (i.e., test A was the pre-test, B the immediate post-test, test C the delayed post-test during month one, and in month two test B was the pre-test, C the immediate post-test, and test A the delayed post-test, etc). All participating students in a month took the same pre-test, post-test and delayed post-test. We administered a survey to all students at the end of the rotation along with the delayed post-test.

Tests that categorized discordant and concordant groups from February 2016 to September 2016 (Figure) were analyzed based on the group assigned and subject of questions. (I.e*.,* the concordant group included the fever group’s performance on fever questions and SOB group on SOB questions. The discordant group included the fever group’s performance on SOB questions and the SOB group on fever questions.) Each subject contributed one set of concordant results and one set of discordant results.

Study participation was voluntary and anonymous. The study was approved by the University of Southern California’s Institutional Review Board. Students reported their specialty of interest via declaration in the visiting student application service application as well as in a post-rotation anonymous survey. Only months in which all students stated an interest in EM and months in which no students stated an interest in EM were used for analysis of completion rates (see Table 1). We queried the Zaption system for monthly reports on the number of video views and total minutes of video watched. We identified unique views by the student’s anonymous viewing “name,” which was required to view the video. Views that lasted only seconds were not counted.

### Key outcome measures

The primary outcome measured was the difference in improvement of test scores from pre-test to immediate post-test between test questions concordant with the video(s) viewed and test questions discordant with video(s) viewed (e.g*.,* fever questions from participants watching the fever video vs. fever questions for participants watching the SOB video). For purposes of data analysis, we used an intent-to-treat model with participants analyzed as assigned, regardless of whether they viewed the video or not. The secondary outcome measures included overall improvement in scores and difference in scores between the pre-test and delayed post-tests, both overall and within concordant and discordant groups.

In the second portion of analysis, the outcome measure was completion of the video. The variables evaluated were specialty of interest (EM vs. other specialty) and time of distribution of the lecture links (two weeks vs. three days).

### Data analysis

We analyzed the percent questions correct and change in percent questions correct between concordant groups as well as discordant groups. Because the tests were taken anonymously the unit of measurement was total change per group per month. The difference was analyzed for significance using the Mann-Whitney U test per the small sample size.

We determined medical student lecture viewing by the Zaption video logs. We reviewed the log during the time period from lecture link distribution to in-class session.

Using a chi-square statistic, we compared the difference in number of students viewing the lectures who were EM bound with those who were not. A similar comparison with chi-square analysis was made between students who viewed the lectures with three days’ notice vs. two weeks’ notice.

## RESULTS

In the analysis of concordant scores, the pre-test averaged 56.7% correct, the immediate post-test averaged 78.1% correct, and the delayed post-test was 67.2%. In the discordant groups, the pre-test averaged 51.9%, the immediate post-test averaged 67.1% correct, and the delayed post-test was 68.8%. In the concordant groups, the immediate post-test scores improved by 21.4%, compared with 15.2% in the discordant groups (p = 0.66). In the delayed post-test the concordant scores improved by 10.5% and discordant scores by 16.9 percent (p=0.61). (See Table 2 and sample test supplemental material A.)

We queried students via an anonymous survey regarding their engagement with the videos, the case discussion and their preferred format (supplemental material B). Surveys were collected for nine months of data collection (two months more than test data collection). We collected 97 surveys. When asked, “how engaged are you with a traditional lecture format?,” students answered a median of 5.3 on a seven-point Likert scale (where 1 indicated “not engaged” and 7 indicated “highly engaged”). The students reported a median of 5.0 engagement with the online videos. When asked which format they preferred, 34 students (35% who completed the survey) preferred traditional lecture and 60 students (62% surveyed) preferred the combined format of pre-class online videos with in-class case discussion. (See discussion below regarding this apparent conflict of results on the survey).

Specific comments by students regarding the two educational formats and the flipped classroom method are summarized in supplemental material C.

In the 15-month study period, 64% of students rotating through the EM elective viewed the priming videos (Table 1). During the 10 months in which exclusively EM-bound students were rotating (n=144), 71.5% (47.1–92.9) of students viewed the lectures. In the four months in which students were not EM-bound (n=54), 55.6% (33.3–71.4) of students viewed the lectures (Table 3a, p=0.033).

Participation was 60.2% (33.3–92.9) when lecture links were given three days in advance and 68.7% (47.1–98.9) when links were given two weeks in advance (Table 3b; p=0.197). Video viewing time averaged >90% of total video length.

## DISCUSSION

The flipped classroom has been used in many educational venues.^3^,^4^,^7^–^13^ While learner experience has been favorable, few data are available on its effectiveness in medical education.^1^,^2^,^4^,^14^ Proving the flipped classroom’s effectiveness is challenging, as the details of implementation are not well defined. This study evaluated the effectiveness of priming on a topic with the students serving as their own control, as they were tested on two topics and only primed on one. Post-test scores improved, but there was not a statistically significant difference in scores on the students’ “primed” topic. This may be because either priming may not significantly impact learning or the tests were too short to determine a difference.

The flipped classroom’s success correlates with learner participation. This study found a statistical difference in video completion rates of EM-bound students compared to students pursuing other specialties. Completion rates of preparatory materials by students using the flipped classroom have been reported with varying degrees of participation;^1^,^5^ and literature is sparse regarding engagement and/or participation of medical students based on their planned specialty. In this study, the preparatory material for the flipped classroom was optional and in addition to the required learning for the clerkship. Despite this fact, the majority (64%) of students viewed the videos prior to class. This indicates an overall favorability to electronic-enhanced education.

Preparatory material must be completed prior to the in-class session in the flipped classroom. One week in advance may be the ideal time to distribute materials, but a definitive answer is not yet known.^16^,^17^ This study demonstrated that three days’ notice appears to be adequate for distribution of materials for the flipped classroom, as we found no statistical difference in completion rates between groups that received three days’ vs. two weeks’ notice to view the videos.

Learners reported a high satisfaction rate with the flipped classroom model and appreciated the flexibility and efficient presentation of material.^1^–^6^,^18^ We posit that because EM was the chosen field of EM-bound students this likely increased clinical relevance and significantly contributed to the fact that they more commonly viewed the lectures compared to peers pursuing other fields.

The ideal format of preparatory materials and quantity of material to be prepared is not yet known in flipped- classroom implementation.^19^–^23^ Approximately 60 minutes of preparatory time has been recommended but not rigorously evaluated, particularly in medical student education.^16^,^17^,^24^ Online videos were used in this study as video learning has been demonstrated to be effective.^20^,^25^ However, the ideal format of electronic education is still unknown.^19^,^21^,^22^,^26^,^27^ Interpolated questions were not used in this study but have been previously shown to promote superior knowledge retention and learner engagement with the material.^1^,^28^ Online educational methods particularly appeal to the millennial generation, which is less tolerant of a traditional lecture format.^13^,^14^,^29^ Additionally, adult learners tend to prefer independence, freedom and flexibility in their learning environment.^30^

Surveyed students reported being more engaged with a “traditional lecture” rather than the online video, but the majority answered in a different question that the method of priming online videos was overall preferred. A possible explanation for this apparent contradiction is that the distributed survey asked a question that was not clear to the students regarding the traditional lecture. Some students interpreted it to ask about the interactive case session with the faculty and others interpreted it to mean a “traditional” didactic lecture that was not interactive or case-based. Multiple students commented that they felt more prepared for the session and were able to interact with the material better after watching the video. They also appreciated that they could view the videos on their own time and at their own pace. They felt more prepared for the case discussion and could ask clinically relevant questions of a more complex nature. (See specific student comments in supplemental material C.)

In this study overall, 64% of students viewed the lectures prior to the class session. This completion rate is similar to other reported flipped-classroom participation.^31^ Accountability in the implementation of the flipped-classroom model may be an issue and impact its effectiveness. If essential didactic material is to be covered, students must participate and prepare prior to class time. Self-reported completion rates may not match actual completion rates when video viewing logs are reviewed.^1^

## LIMITATIONS

There were several limitations to this preliminary and descriptive study. Primarily, the preparatory material in a flipped classroom is typically mandatory. In this study, to avoid a sense of coercion to participate in research the video material was optional. Viewing rates may have increased if they were required. Additionally, no subject number was given to each student. It is possible that a single student logged onto the system using a different name and viewed the material more than once but was counted as an additional student viewing the videos. It is also not known if the student actually watched the video or merely let it play while engaging in other activities. Interpolated questions within the videos may have increased learner engagement and promoted more active learning during video viewing. Tracking video view times and interpolated question responses may add to the value of the priming videos and increase the success of the flipped classroom.

The association between material completion and interest in EM is assumed to have occurred because the material matched the students’ field of interest, but this may be due to increased level of compliance overall by EM-bound students. Similar studies in other core and elective rotations would be needed to verify causality. The time of year (and relation to match- and rank-list submission) may also play a role in medical student participation. This was not examined in this study. Additionally, there may be a Hawthorne effect of students participating in a study. They may have participated more and been influenced to prefer the format. This study was also performed at a single site and may not be generalizable to other institutions.

Despite gathering test data for seven months with nearly 100 students, the sample size may not have been large enough to show a difference on test scores. Additionally, each test was six questions and likely of varying degrees of difficulty. The tests used to evaluate learning were peer reviewed but not validated prior to the study, which limits the power of results. Test validity is a challenge in assessing knowledge.^32^ This may account for the lack of statistical significance between the concordant and discordant groups. The survey was also not piloted prior to study administration. Additionally, in an attempt to facilitate ease of participation and preserve anonymity in test administration, no subject number was given to each student. Therefore, we were not able to make a direct comparison of pre-test to post-test scores, which may also have limited statistical significance.

## CONCLUSION

Immediate post-tests and delayed post-tests improved, but priming was not demonstrated to be a statistically superior educational method in this study. Though the students’ concordant test scores were not statistically superior, the preference of surveyed students was to be offered additional priming material prior to an interactive case discussion. Medical student completion of the preparatory materials increased when rotating students were EM bound. Participation rates were not significantly different when lecture links were distributed at two weeks vs. three days, although there was a trend toward greater viewing compliance when links were sent out two weeks in advance.

## Supplementary Information







## Figures and Tables

**Figure:**
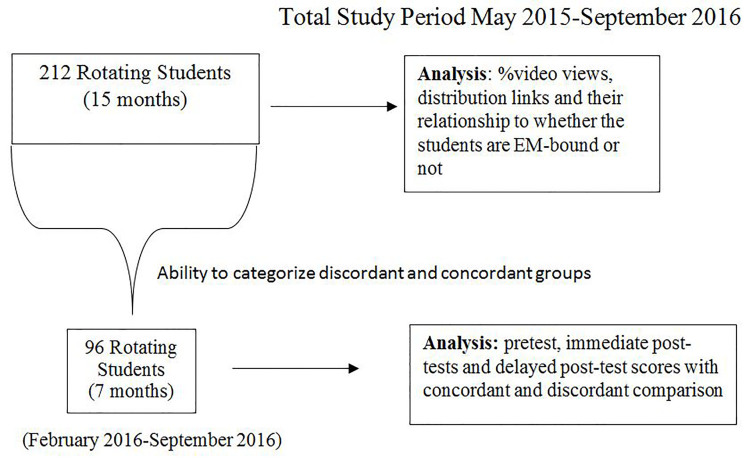
Inclusion period for a study of the “flipping classroom” educational method with 212 students rotating in emergency medicine from May 2015 – September 16

**Table 1:** Rotation student characteristics, time of material distribution, video views and average immediate post-tests.

Rotation date	Total rotating students (#)	EM-bound (#, [%])	KSOM (#)	Visiting students (#)	Video links prior to class	Video views* (#, [%])	Average immediate post-test score (%)
5/18–6/14/2015‡	17	17 (100)	17	0	2 weeks	8 (47.1)	----------
6/29–7/26/2015‡	13	13 (100)	12	1	3 days	7 (53.8)	----------
7/27–8/23/2015‡	15	15 (100)	0	15	3 days	8 (53.3)	----------
8/24–9/20/2015‡	14	14 (100)	0	14	3 days	12 (85.7)	----------
9/21–10/18/2015‡	14	14 (100)	0	14	3 days	11 (78.6)	----------
10/19–11/16/2015‡	14	14 (100)	0	14	3 days	13 (92.9)	----------
11/16–12/13/2016	14	2 (14.3)	12	2	3 days	3 (21.4)	----------
1/4–1/31/2016‡	15	0 (0)	14	1	3 days	5 (33.3)	----------
2/1–2/28/2016†‡	14	0 (0)	13	1	3 days	9 (64.3)	63.5
2/29–3/27/2016†‡	14	0 (0)	14	0	2 weeks	10 (71.4)	79.6
3/28–4/21/2016†‡	11	0 (0)	11	0	2 weeks	6 (54.5)	81.8
5/16–6/12/2016†‡	14	14 (100)	13	1	2 weeks	10 (71.4)	48.9
6/27–7/24/2016†‡	15	15 (100)	15	0	2 weeks	8 (53.3)	69.3
7/25–8/21/2016†‡	14	14 (100)	0	14	2 weeks	13 (92.9)	74
8/22–9/18/2016†‡	14	14 (100)	0	14	2 weeks	13 (92.9)	81.6

*EM*, emergency medicine; *KSOM*, Keck School of Medicine of the University of Southern California.

* Number of video views defined as unique views; the average viewing length was >90% of total video length.

† Months in which tests results were analyzed in concordant and discordant groups.

‡ Months in which students were exclusively EM-bound, or all students were not planning to pursue EM.

--------- Scores not evaluated due to inability to match concordant and discordant groups.

**Table 2:** Average test scores for concordant and discordant groups.

Group	Pre-test (%)	Immediate post-test (%)	Percent difference from pre-test	Concordant v. discordant‡	Delayed post-test (%)	Percent difference from pre-test	Concordant v. discordant‡
Concordant*	56.7 (35.7–75.4)	78.1 (54.5–87.5)	21.4	P=0.66	67.2 (56.3–87.5)	10.5	P=0.61
Discordant†	51.9 (42.9–77.2)	67.1 (50.9–83.3)	15.2		68.8 (55.4–86.1)	16.9	

* Concordant group: performance on fever questions by those who were in the fever group and performance on shortness of breath (SOB) questions by those who were in the SOB group

† Discordant group: performance on SOB questions by those who were in the fever group and performance on fever questions by those who were in the SOB group

‡ P-values calculated difference between concordant vs. discordant scores using Mann-Whitney U statistics

**Table 3a:** Video viewing based on future specialty and time to dissemination of lecture links: The number of students who viewed optional educational online lectures prior to class session.

	EM-bound students (144)	Non-EM bound students (54)	p-value
Viewed	103	30	
Not viewed	41	24	0.033

*EM*, emergency medicine.

**Table 3b:** Time to dissemination: The number of students who viewed lectures with three-day and two-week notice (EM and non-EM bound combined).

	3 day (n=113)	2 weeks (n=99)	p-value
Viewed	68	68	
Not viewed	45	31	0.197

*EM*, emergency medicine.
