# Supplementary material for: Flipping the Classroom in Medical Student Education: Does Priming Work?
Source: West J Emerg Med. 2017 Dec 5;19(1):93–100. doi: 10.5811/westjem.2017.8.35162 (PMC5785208; doi:10.5811/westjem.2017.8.35162)
Supplement: Supplementary file 1 [file wjem-19-93-s001.docx]

TEST C

1. What is currently the most common serious bacterial illness in young infants?

a. Cellulitis

b. Meningitis

c. Pneumonia

d. Urinary tract infection

2. Which of the following is the recommended method for confirming a urinary tract infection in a febrile infant?

a. Bag urine with urine dipstick

b. Bag urine with urinalysis

c. Catheterized specimen with urinalysis

d. Catheterized specimen with urine culture

3. Which of the following is generally TRUE regarding mild croup in the pediatric patient?

a. Albuterol is indicated

b. Oral dexamethasone is indicated

c. Racemic epinephrine is indicated

d. Stridor is present at rest

4. Which of the following is TRUE regarding the diagnosis and evaluation of croup in the pediatric patient?

a. Consult ENT for laryngoscopy, if stridor is present

b. Order neck xray to evaluate for steeple sign in all croup cases

c. Perform nasopharyngeal swab to confirm via rapid ELISA test

d. No diagnostic testing is needed

5. Which of the following statements is TRUE regarding pediatric patients with acute bronchiolitis?

a. Can require intubation

b. Associated pneumonia is common

c. Occurs in patients >2 years of age

d. Peak months are October and November

6. Which of the following is the best diagnostic test for bronchiolitis?

a. Chest x-ray

b. Physical exam

c. Vital signs

d. Viral Panel

7. Which lecture did you view?

a. Fever

b. Shortness of Breath

c. I was assigned Fever but did not view

d. I was assigned SOB but did not view
