# Supplementary material for: Flipping the Classroom in Medical Student Education: Does Priming Work?
Source: West J Emerg Med. 2017 Dec 5;19(1):93–100. doi: 10.5811/westjem.2017.8.35162 (PMC5785208; doi:10.5811/westjem.2017.8.35162)
Supplement: Supplementary file 2 [file wjem-19-93-s002.docx]

**Thank you for participating in our study. Help us gather further data and more effectively educate students in the future by answering the following questions:**

**1. For the traditional lecture, how engaged were you (circle one)?**1 (not engaged at all) 2 3 4 (met the objectives) 5 6 7 (extremely engaged)

**2. For the self-directed video and interactive case discussion, how engaged were you (circle one)?**1 (not engaged at all) 2 3 4 (met the objectives) 5 6 7 (extremely engaged)

**3. Which of the following learning formats did you prefer?**A. Traditional lecture
B. Self-directed video + interactive case discussion
**Why?** (enter text below)

**4. What percent of the assigned video lecture did you complete? (Out of 100%)**

**5. To which specialty are you applying?**
