# Supplementary material for: Flipping the Classroom in Medical Student Education: Does Priming Work?
Source: West J Emerg Med. 2017 Dec 5;19(1):93–100. doi: 10.5811/westjem.2017.8.35162 (PMC5785208; doi:10.5811/westjem.2017.8.35162)
Supplement: Supplementary file 3 [file wjem-19-93-s003.docx]

**Supplemental material C: Sample survey comments from students**

| Comments in support of video learning: | The video provided concrete, clearly organized facts + the interactive case gave the opportunity to apply knowledge  I felt more prepared for the discussion after having watched the video  I found it helpful to watch the video first so as to have a background knowledge for the lecture.  I felt more prepared for the discussion after having watched the video  It is easier to process information in a self-directed video. I can stop or go back if I don’t understand a concept.  A self-directed video let me re-watch portions that I needed clarification on  The videos were more to the point and shorter  The video information was presented in the format of common clinical scenarios, questions and misconceptions  I can play the videos at a higher speed and stay more focused for a shorter period of time.  I can focus easier watching a video versus in the classroom. There are more distractions.  I appreciated the freedom to watch the video on my own time and pause the video so I could recap the material. |
| --- | --- |
| Comments in support of a traditional lecture | Physically being at a lecture in personal (and with a good lecturer) keeps me more engaged  I like a lecture format (over videos) for a more personal interaction  Just a lecture requires less total time |
| Comments in support of utilizing the flipped classroom | I prefer [completing pre-class video preparation] because it sticks better in my head since we go over material twice. However it does take more time and is less efficient. Also, I can pause and rewind if I didn't catch something or need to review the material.  I enjoyed how interactive the case discussion was. I also find video helpful for learning.  I prefer the format of watching a video followed by a case discussion. It is much more engaging and promotes active learning  The in-class case discussion was more interactive and I was able to think about the cases in more depth  The case discussion allowed opportunities for questions and clarification. It was interactive and fun, more personal  The case discussion format provides more personal interaction. This human interaction keeps our interest/focus and allows the student to ask more questions  Interactive cases better applied the knowledge.  All things interactive are better than traditional lecture. It is useful to have a starting knowledge base (via the videos) before engaging with faculty in discussion of specific cases.  The video + case discussion gave an opportunity to have a greater interaction with the attending and it was more applicable to how we approach real cases in the emergency department.  I like to review material prior to a lecture; it helps me to stay engaged and better solidify information. It is a good primer to help follow the in-class discussion. It is ultimately a better use of my time. I am overall more actively engaged |
